# Associated factors of barriers to help-seeking among postpartum women with and without (childbirth-related) posttraumatic stress disorder: results from the cross-sectional study INVITE

**DOI:** 10.1186/s12884-025-07933-1

**Published:** 2025-11-27

**Authors:** Marlena Sophie Harder, Lara Seefeld, Julia Schellong, Susan Garthus-Niegel

**Affiliations:** 1 Institute and Policlinic of Occupational and Social Medicine, Faculty of Medicine, TUD Dresden University of Technology, Dresden, Germany; 2 Department of Psychotherapy and Psychosomatic Medicine, Faculty of Medicine, TUD Dresden University of Technology, Dresden, Germany; 3 Department of Psychology and Neuroscience, Dalhousie University, Halifax, Canada; 4 Institute for Systems Medicine (ISM), Faculty of Medicine, Medical School Hamburg, MSH, Hamburg, Germany; 5 Department of Childhood and Families, Norwegian Institute of Public Health, Oslo, Norway

**Keywords:** Postpartum, PTSD, CB-PTSD, Barriers to help-seeking, INVITE study

## Abstract

**Background:**

Mental health disorders are widespread during the postpartum period, yet only a minority of affected women seek professional help for their symptoms. While prior studies have explored barriers to help-seeking, certain factors yielded inconsistent results. Our study aimed to investigate whether these factors do not serve as barriers themselves, but rather as predictors of barrier formation. Given the limited previous research on postpartum women affected by posttraumatic stress disorder (PTSD), we examined those affected by childbirth-related PTSD (CB-PTSD) and general PTSD (gPTSD).

**Methods:**

We used data from the cross-sectional study INVITE (INtimate partner VIolence care and Treatment prEferences in postpartum women). For this study, a total of *N* = 3,874 postpartum women were inquired about their mental health status and barriers keeping them from seeking help. We categorized these women into three distinct groups: (1) women affected by CB-PTSD, (2) women affected by gPTSD, and (3) non-affected women. For each group, we conducted multiple linear regression analyses to examine whether certain factors (self-report of PTSD, knowledge of healthcare services, previous help-seeking, social support, severity of symptoms, and household net income) are associated with previously identified barriers to help-seeking.

**Results:**

Our findings revealed that higher social support predicted lower barriers to help-seeking, particularly among women with gPTSD. Self-reporting PTSD predicted lower barriers among women with CB-PTSD. Previous help-seeking predicted lower barriers among women with CB-PTSD, but higher barriers among those with gPTSD. Greater knowledge of healthcare services predicted lower barriers for non-affected women, but higher barriers for women with CB-PTSD. A higher household net income predicted lower barriers only among non-affected women. We found no association between symptom severity and barriers to help-seeking.

**Conclusions:**

High levels of social support and self-report of postpartum PTSD emerge as crucial elements in reducing women’s barriers to help-seeking. Strengthening these factors can be achieved by providing psychoeducation to women and their social surroundings, equipping them to identify symptoms and respond accordingly. Moreover, implementing PTSD screenings for all postpartum women may facilitate symptom recognition, thereby reducing barriers to help-seeking. Additionally, educational campaigns could help to reduce stigmatization and shame associated with postpartum mental health disorders within the society, fostering greater understanding and empathy for affected women.

## Background

### Posttraumatic stress disorder in the postpartum period

**Fig. 1:**
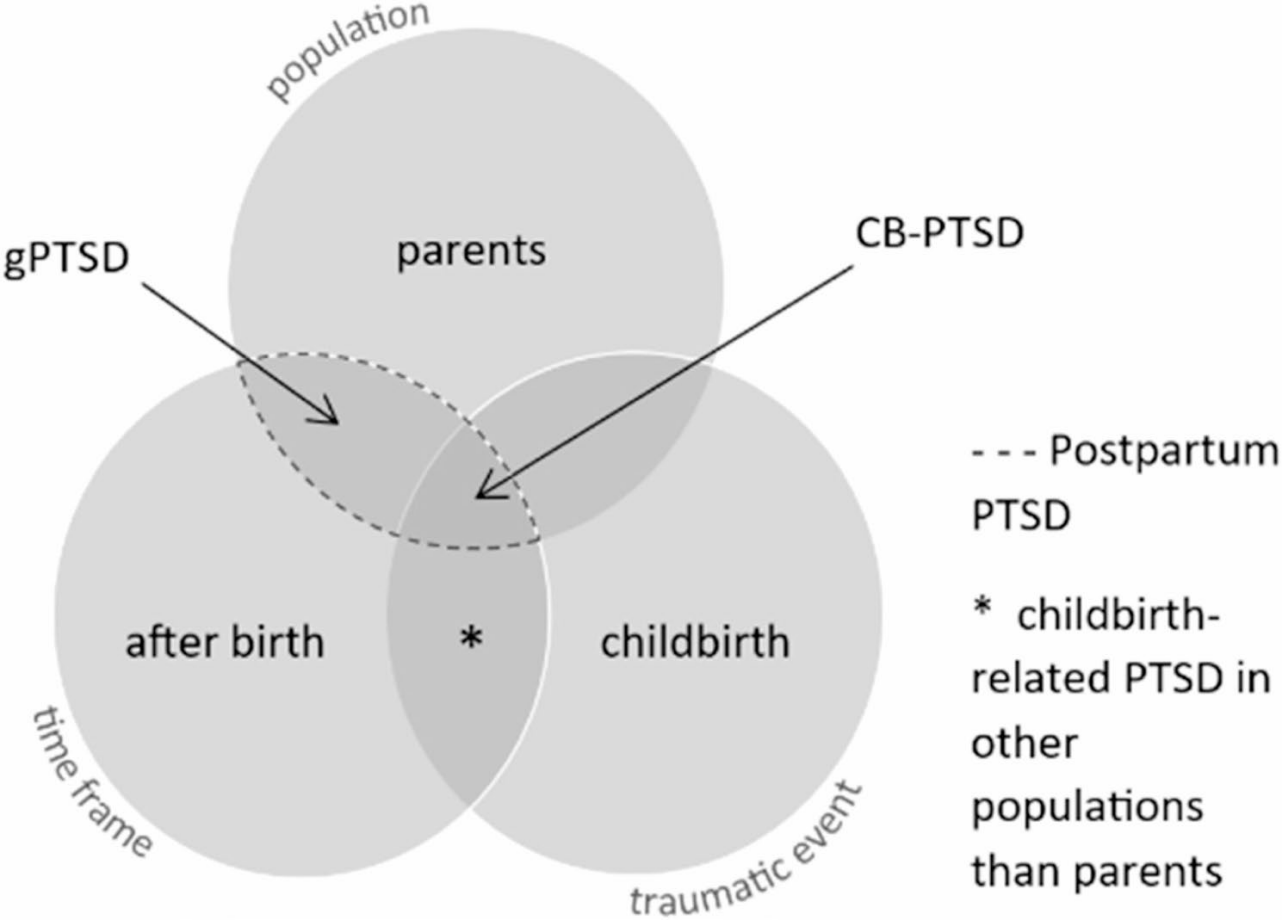
Terminology of Postpartum PTSD. Definitions and abbreviations used in this work for the symptom groups of childbirth-related PTSD (CB-PTSD) and general PTSD (gPTSD), collectively referred to as postpartum PTSD. Adapted from Heyne et al. [4]

‘The first few months were awful. I hit the nail on the head when he was about two months old as I needed to put distance between me and it because for a while it was all I could think of, proper having flashbacks’ [1]. This is a quote from a woman experiencing symptoms of posttraumatic stress disorder (PTSD) in relation to childbirth [1]. Symptoms of PTSD include intrusions or re-experiencing, avoidance, negative alterations in mood or cognition, and increased hyperarousal following the experience of a traumatic event. The traumatic event could involve an actual or threatened death, serious injury, or sexual violence. Exposure may have been direct, witnessed, or indirect [2]. Regarding PTSD during the postpartum period, the underlying traumatic event could have been childbirth itself or a different index event unrelated to childbirth [3]. In this paper, we will assess both cases and utilize the term childbirth-related PTSD (CB-PTSD) solely for women who have encountered childbirth as the primary traumatic event and general PTSD (gPTSD) for postpartum women who have experienced a different traumatic event. Postpartum PTSD will refer to both groups of women (see Fig. 1) [4].

According to the American Psychiatric Association [2], symptoms must persist for at least one month and have a substantial impact on functioning for a PTSD diagnosis. About 5.44% of women meet these criteria for postpartum PTSD [5] and about 4.7% of women for CB-PTSD [6]. Prevalence rates as high as 15.7–18.5% are common among high-risk groups for postpartum PTSD characterized for instance by preterm birth or pregnancy complications [5, 7]. There is no prevalence rate identified for women with gPTSD, as prior research has primarily focused on women with CB-PTSD rather than those with gPTSD, as outlined in the following section.

### Impact of postpartum PTSD

To date, research has only partially distinguished between CB-PTSD and gPTSD [5, 6, 8]. CB-PTSD has been found to have harmful effects, negatively impacting a child’s development [9, 10], couple relationship satisfaction [11], as well as the overall well-being of the family [12–14]. Symptoms of gPTSD may adversely impact women’s relationships, their pregnancies, and birth outcomes [15–18]. Additionally, it may affect infants’ emotion regulation and development [5, 19, 20].

**Fig. 2:**
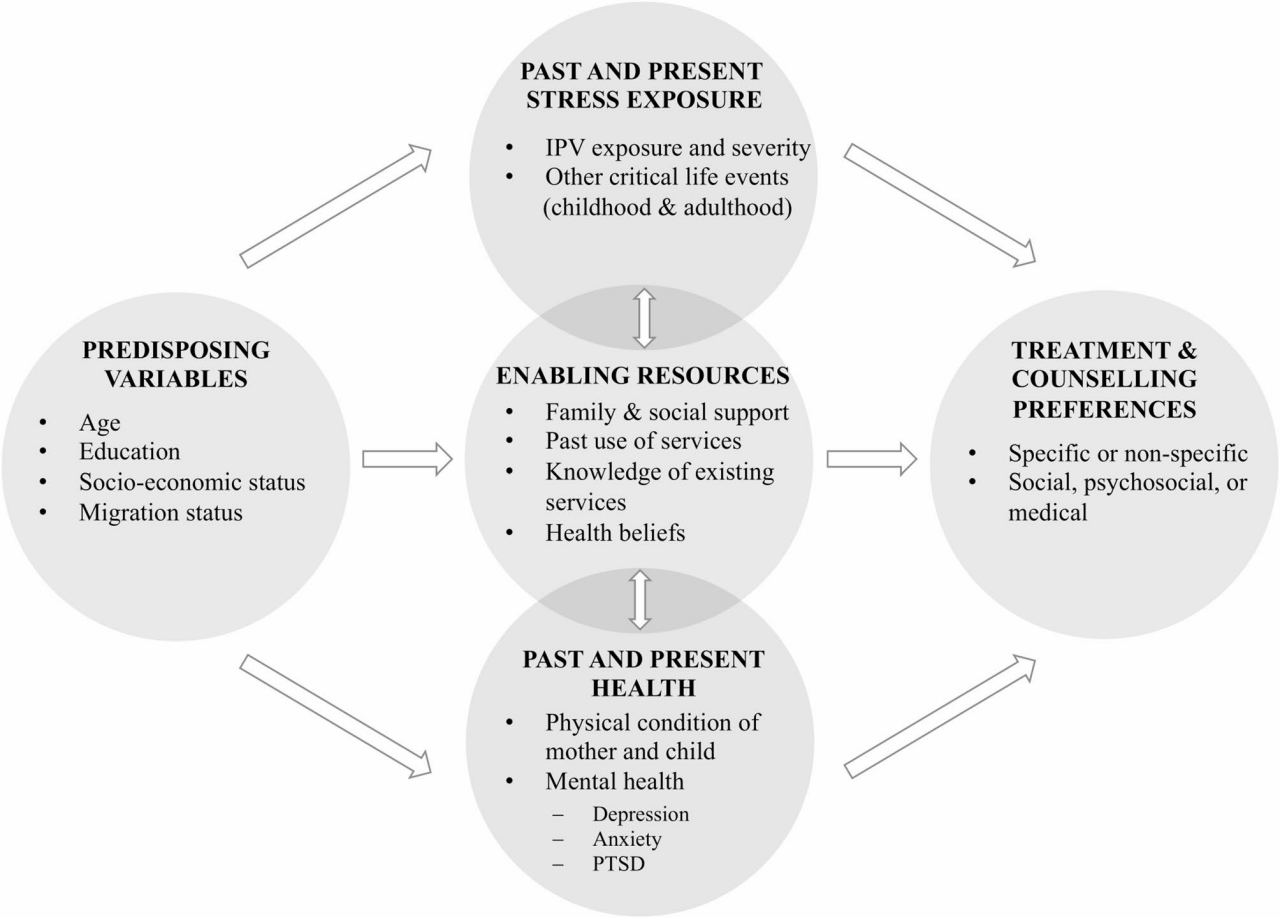
The Theoretical Model of Help-Seeking (Seefeld et al.,* 2022)*. Theoretical framework of the INVITE study. Adapted by Seefeld et al. [27] from Andersen [28] and Liang et al. [6]

There is ongoing research on professional treatment for postpartum PTSD. First effective methods for CB-PTSD have been identified, such as the combination of a visuospatial task and trauma-related cues [21, 22]. Among women with gPTSD, preliminary findings indicate the efficacy of written exposure therapy in reducing symptoms [23]. In the general population, cognitive behavioral therapy has been identified as an effective intervention for individuals with PTSD [24]. Furthermore, numerous healthcare services are accessible for postpartum women, such as family midwives and counseling centers. Nevertheless, only a minority of affected women seek professional help for their symptoms [25, 26]. This tendency can be attributed to various factors, as illustrated in the theoretical model of help-seeking (Fig. 2) proposed by Seefeld et al. [27].

### Barriers to help-seeking

The theoretical model of help-seeking [27] combines two models of help-seeking [4, 28], adapted for motherhood-related factors. The model illustrates predisposing variables that predict previous and current stress exposure, enabling resources, and health. These three factors are related bidirectionally and in turn predict treatment and counselling preferences. This suggests that specific resources can aid postpartum women in help-seeking; conversely, the lack of such resources can act as a barrier to help-seeking [27]. This is in line with previous findings highlighting the different reasons why women experiencing mental health problems in the postpartum period do not seek help [29]. These reasons include for example fear of stigmatization [30, 31], fear regarding treatment characteristics (e.g., being judged by health professionals) [32], health beliefs (e.g., believing that symptoms are a normal part of motherhood) [33], and instrumental barriers (e.g., no childcare) [34]. However, these factors have mainly been investigated in relation to postpartum depression disorder (PPD) or postpartum anxiety disorder (PAD), with limited attention to the specific barriers that women with postpartum PTSD encounter. Therefore, it is imperative to conduct further research on women with postpartum PTSD.

Moreover, while prior studies have examined barriers to help-seeking, certain factors yielded inconsistent results. This study aims to investigate whether these factors do not serve as barriers themselves, but rather as predictors of barrier formation. For instance, insufficient knowledge of available healthcare services may predict a higher fear of treatment. Thus, increasing knowledge of services may reduce that fear. Identifying such predictors can help inform governments and healthcare services which steps need to be taken to overcome barriers to help-seeking among women with and without postpartum PTSD. Potential predictors of barriers are discussed in the following section.

### Predictors of barriers to help-seeking

First of all, a woman must recognize that a certain problem exists, in order to act and seek help [35]. Lacking recognition may lead to the misinterpretation of PTSD symptoms as being a normal part of motherhood, which do not need further attention [26, 36]. Moreover, many women who have recognized the problem may not seek help, because they do not know which services are available or where to go to [26, 37]. Additionally, help-seeking behavior may be influenced by previous experiences with healthcare services. According to Watson et al. [38], previous positive experiences with help-seeking predict the likelihood of seeking help again, while negative experiences may deter individuals from further help-seeking. Furthermore, a supportive social environment is crucial to encourage professional help-seeking among postpartum women [3, 33]. However, the social circle may also normalize existing PTSD symptoms, discouraging women from help-seeking [29, 36]. Besides, the severity of symptoms may affect help-seeking behavior. A meta-ethnography examining women with PPD suggested that current depressive symptoms keep women from seeking help due to a lack of energy [39]. Whether this applies to women with postpartum PTSD as well remains unclear. Among veterans with PTSD, higher symptom severity was associated with a higher likelihood of seeking help [40]. Smith et al. [40] suggested that some individuals may only acknowledge their need for help when experiencing highly severe symptoms, while others with such symptoms may not be willing to undergo treatment due to concerns about their emotional stability when discussing the traumatic event. Consequently, the impact of symptom severity on help-seeking behavior remains ambiguous, especially among women with postpartum PTSD. Women’s help-seeking behavior may also be affected by their household net income, such as the ability to afford transportation for attending treatment sessions. We infer from Webb et al.’s [33] findings that economic status, household net income, and healthcare costs are instrumental factors creating barriers to mental healthcare services.

As shown, previous research indicates several potential predictors of barriers to help-seeking among postpartum women. However, the majority of research has focused on women with symptoms of PPD or PAD, with only few studies examining women with postpartum PTSD [3]. Therefore, it is crucial to investigate such predictors among these women. Likewise, it is important to examine women who are not affected by PTSD, as they may serve as a control group by experiencing subclinical symptoms of PTSD that require support.

For these reasons, the present study aims to determine predictors of barriers to help-seeking among women with and without postpartum PTSD by addressing the following research questions:

1. To what extent do factors such as self-report of PTSD, knowledge of healthcare services, previous help-seeking, social support, severity of symptoms, and household net income predict barriers to help-seeking among postpartum women affected by CB-PTSD, gPTSD, comorbid CB-PTSD and gPTSD, and among non-affected women?
2. Do these factors differ among postpartum women affected by CB-PTSD, gPTSD, comorbid CB-PTSD and gPTSD, and non-affected women?

## Methods

### Design

To investigate these research questions, data from the cross-sectional study INVITE (‘INtimate partner VIolence Treatment prEferences’) were used. The INVITE study aims to examine women affected by PPD, PAD, postpartum PTSD, and intimate partner violence, to identify their preferences and barriers to treatment and counselling. The recruitment included all maternity hospitals and most birth centers in Dresden. All approached women who lived in and around Dresden, Germany, and had sufficient German or English language skills were eligible to participate between 6 weeks and 6 months postpartum. Using structured telephone interviews, data were collected by trained student assistants. The interviews were quantitative in nature, with self-reported measures being read out to the women. As an incentive for participation, all women received 20€ [27].

Data collection and management was facilitated using Research Electronic Data Capture (REDCap), a secure, web-based application for data capture as part of research studies, hosted at the ‘Koordinierungszentrum für Klinische Studien’ at the Faculty of Medicine of the Technische Universität Dresden [41].

### Sample

**Fig. 3:**
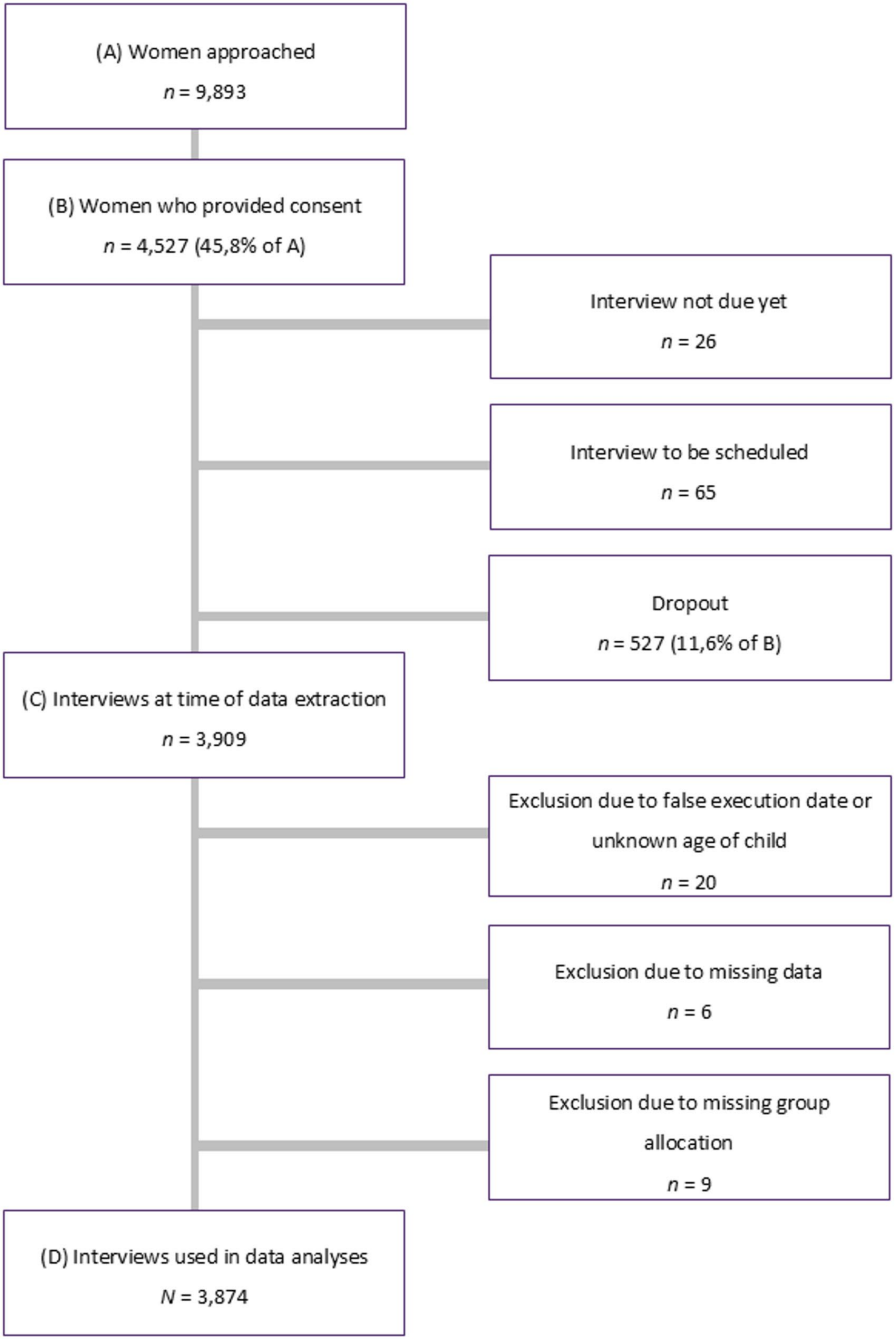
Flowchart of the INVITE Study. Illustration of the response rate, dropouts, exclusions, and final sample size based on the recruitment process of the INVITE study between November 2020 and July 7^th^, 2023

This paper used data from Version 4 of the quality-assured datafiles, collected between the study initiation in November 2020 and July 7th, 2023. Figure 3 shows that out of the 9,893 women approached, 4,527 (45.8%) provided written informed consent to participate. During the data extraction process, 26 women had not yet been interviewed due to their childbirth occurring outside the requisite six-week window, and 65 women because they had not yet been reached for the scheduled appointment. Furthermore, 527 women dropped out of participation because they could not be reached for the interview within the time frame or because they revoked their consent. Of the 3,909 interviews, those conducted prior to six weeks postpartum or after 6 months postpartum, as well as those with incomplete data regarding the date of childbirth, were excluded from further analyses (*n* = 20). In addition, six participating women were excluded due to missing data across all variables of interest. Nine women also had to be excluded due to missing values in group-specific items, resulting in an incomplete group allocation. Therefore, our data analysis was based on a total sample of *N* = 3,874 women.

### Materials

#### Group Variables

To assess CB-PTSD, the German version of the City Birth Trauma Scale (City BiTS) [42, 43] was used. The questionnaire contains 22 items which align with the diagnostic criteria for PTSD as per DSM-5 [2]. To identify diagnostic criterion A items (e.g., perceived threat of (the child’s) death or serious injury), a yes/no-scale was used. Other criteria comprise intrusions, avoidance, negative cognition and mood, as well as hyperarousal. Women were asked about the onset of symptoms (before or after childbirth). Symptoms’ frequency over the past week was rated on a Likert-type scale of 0 (‘not at all’) to 3 (‘5 or more times’). The score ranges from 0 to 60 with a higher score indicating a greater severity of symptoms [42]. Additionally, women’s distress and disability were rated as yes, no, or sometimes.

To measure gPTSD, a shortened version of the Primary Care PTSD Screen for DSM-5 (PC-PTSD-5) [44] was used. To ensure the assessment of potential PTSD symptoms among all women in our sample, an adjustment to the screening instrument was made in the INVITE study. A prompt was included for women to recall an event that was ‘so frightening, horrible, or upsetting’ that they may have experienced any of the following symptoms during the past month [27]. These symptoms include intrusions, avoidance, hypervigilance, feelings of detachment, and negative cognitions. Women were also asked to rate their level of stress for each symptom using a five-point Likert scale ranging from 0 (‘not at all’) to 4 (‘extremely’). The score ranges from 0 to 20, with a higher score indicating a greater severity of symptoms. Finally, women were asked to describe the type of traumatic event they had experienced, using the trauma list from the Posttraumatic Diagnostic Scale for DSM-5 [45]. The category ‘other traumatic event’ allowed for open-ended responses.

#### Outcome

To assess barriers to help-seeking, a questionnaire based on the Health Belief Model [46] was designed by Seefeld et al. [27]. The questionnaire contains 15 items representing different barriers to help-seeking. All participating women were asked how likely each of these barriers would prevent them from seeking help, using a five-point Likert scale ranging from 1 (‘not true at all’) to 5 (‘very true’). Barriers were presented to all women, asking non-affected women to imagine experiencing symptoms of postpartum mental health problems. The total amount of barriers was calculated as the sum score of all responses, with a possible range from 15 (indicating no barriers) to 75 (indicating high barriers). In our sample, the resulting 15 items demonstrated a good internal consistency (Cronbach’s Alpha = 0.80). Based on a factor analysis by Seefeld et al. [47] (publication in preparation), these items were divided into three different subcategories of barriers: (1) fear regarding treatment and stigmatization (e.g., ‘I would feel ashamed while discussing my personal problems’), (2) health beliefs (e.g., ‘I should solve my problems alone rather than confiding in a specialist’), and (3) instrumental barriers (e.g., ‘I would not have childcare’). Each subcategory contained five items. Cronbach’s Alpha indicated moderate internal consistency with α = 0.78 for fear regarding treatment and stigmatization, α = 0.64 for health beliefs and α = 0.58 for instrumental barriers. Jehn et al. used the same barriers questionnaire in their analyses of group differences between women with CB-PTSD, gPTSD, and women without postpartum PTSD [48].

#### Predictors

To assess women’s self-report of PTSD and their previous help-seeking behavior, questions from the INTERSECT study [49] were used, alongside self-generated questions. Women were asked to indicate whether they currently have a mental health problem using a yes/no/don’t know scale. If affirmed, they were asked to specify the type of mental health problem. Women who specified PTSD and were identified by our screening instruments were considered to have recognized their PTSD. These questions were presented before women completed the screening instruments (City BiTS, PC-PTSD-5) to exclude the possibility of any priming effects. Furthermore, women who reported a mental health problem were asked to indicate whether they have received or are currently receiving mental health treatment. If they affirmed, they rated their experiences with treatment services on a 4-point Likert scale ranging from 0 (‘inadequate’) to 3 (‘pretty good’).

To assess knowledge of healthcare services, Seefeld et al. [27] generated a questionnaire based on Simhi et al. [50]. The questionnaire contains twenty different healthcare services comprising personal and professional confidants (e.g., family, midwives), psychosocial and communal services (e.g., telephone counseling), medical services (e.g., general practitioner), and psychotherapeutic services (e.g., psychotherapeutic practice). Women rated whether they already knew about the specific service on a yes/no-scale. Of these responses, a total score ranging between 0 and 20 was calculated to indicate women’s overall knowledge of healthcare services.

The level of available social support was acquired using the F-SozU-14 questionnaire, a shortened version of the Social Support Questionnaire [‘Fragebogen zur Sozialen Unterstützung’], developed by Fydrich et al. [51]. Women responded to 14 items (e.g., ‘There are people who take me for who I am without restriction.’) on a 5-point Likert scale ranging from 1 (‘not true’) to 5 (‘completely true’). The amount of social support was calculated as the mean of all responses, with a possible range from 0 (indicating low social support) to 5 (high social support). The scale’s internal consistency was excellent (α = 0.91).

To assess the severity of symptoms, the City BiTS [42, 43] was used for women with CB-PTSD. The sum score of symptoms over the last week ranged from 0 to 60, with a higher score indicating a greater symptom severity. For women with gPTSD, the PC-PTSD-5 [44] was used. Women rated their level of stress for each symptom on a range from 0 to 20, with a higher score indicating a greater symptom severity.

The household net income was derived from Lampert & Kroll [52] and acted as a proxy for the socio-economic status. Women rated their household’s average monthly net income on six different scales: (1) under 1,250€, (2) 1,250€–2,249€, (3) 2,250€–2,999€, (4) 3,000€–3,999€, (5) 4,000€–4,999€, and (6) over 5,000€.

### Data analysis

Data collection and management was facilitated using Research Electronic Data Capture (REDCap), a secure, web-based application for data capture as part of research studies, hosted at the ‘Koordinierungszentrum für Klinische Studien’ at the Faculty of Medicine of the Technische Universität Dresden [27, 41]. The following descriptive and statistical analyses were conducted using IBM SPSS Statistics (Version 27.0.1.0).

To investigate predictors of barriers to help-seeking, we categorized participating women into four distinct groups: (1) women suffering from clinically significant symptoms of CB-PTSD, (2) women with clinically significant symptoms of gPTSD, (3) women who met comorbid criteria of both CB-PTSD and gPTSD, and (4) women non-affected by clinically significant symptoms of postpartum PTSD. To identify the first group, we used the City BiTS [42, 43] and included all women who met the relevant DSM-5 criteria for CB-PTSD, with criterion A being optional. Criterion A refers to the experience of a traumatic event that could involve an actual or threatened death, serious injury, or sexual violence [2]. During the development of the DSM-5 definition of PTSD symptoms, there was a debate about whether the index event was necessary [53]. It is known that a seemingly uncomplicated event without a direct threat can be perceived as traumatic subjectively [54]. In the context of birth trauma, such events may be a lack of support during labour, operative birth, and dissociation [55]. Therefore, we did not require meeting the A criterion for assignment to the CB-PTSD group. As a result, we also expected a larger group of women with CB-PTSD and therefore greater statistical analysis power [48]. The second group was assessed using the PC-PTSD-5 [44] and comprised all women who experienced at least one traumatic event unrelated to childbirth and met at least 4 of the 5 remaining criteria. As with CB-PTSD, fulfilling Criterion A was also optional for gPTSD. The trauma list of the PC-PTSD-5 included an ‘other traumatic event’ category that allowed open-ended responses. All events reported in this category were included in the analysis to ensure comparability with our approach to CB-PTSD and to account for the subjective perception of trauma. The third group comprised women who met diagnostic criteria for both CB-PTSD and gPTSD, whereas the fourth group included all women who did not meet diagnostic criteria for postpartum PTSD.

For all analyses, we carefully checked the relevant data for statistical requirements and outliers. Descriptive statistics were performed on all relevant variables to characterize the study population, including calculating the mean and standard deviation for the sociodemographic variables. If there were missing values of less than 20% in one scale, we replaced them with the woman’s mean value. In the multiple linear regression analyses, the respective outcome variables were checked for requirements as specified. We examined linear relationships between variables and identified outliers in the outcome. Outliers were defined as any values whose studentized excluded residuals were below − 3 or above + 3 and were subsequently excluded from respective analyses. The amount of these outliers varied across different analyses and is described in the results section. Additionally, we ensured that the residuals were independent from one another, indicating no autocorrelation. We further verified the absence of multicollinearity among predictors and heteroskedasticity in residuals. Finally, we validated that residuals followed a normal distribution.

To analyze our data, we first conducted a multiple linear regression analysis for each of the groups to examine whether the following factors predicted the total amount of barriers to help-seeking. Among women with CB-PTSD, gPTSD, and comorbid CB-PTSD and gPTSD, included predictors were self-report of PTSD, knowledge of healthcare services, previous help-seeking, social support, severity of symptoms, and household net income. Among non-affected women, the predictors self-report of PTSD and severity of symptoms were excluded as they were not applicable. The sum score of barriers to help-seeking served as the outcome variable for all groups. Second, we conducted separate multiple linear regression analyses for each subcategory of barriers across the groups to examine the differential impact of the same predictors on specific barrier dimensions. These dimensions served as our outcome, including fear regarding treatment and stigmatization, health beliefs, and instrumental barriers. In response to the first research question, the regression analyses allowed us to determine which of the predictors were significant (*p* <.05) in each group, and to assess the magnitude of their coefficients in all groups. To address the second research question, we compared predictors to barriers between all groups visually.

## Results

### Descriptive statistics

The final sample comprised *N* = 3,874 women, whose descriptive statistics are presented in Table 1. At the time of the interview, the average age of participating women was 32.95 years (*SD* = 4.64), and their babies were 13.18 weeks old (*SD* = 2.71). Most women were born in Germany (91.2%), were in a partnership (97.7%), and reported no current mental health problem (89.3%). On average, women reported a moderate number of barriers to help-seeking (*M* = 31.5, *SD* = 6.98).

**Table 1:** Descriptive statistics of the total sample

Sample Characteristics	Total Sample (*N* = 3874)^a^
**Maternal age**^b^	32.95 ± 4.64 (16.78 - 53.96)
**Child's age**^c^	13.18 ± 2.71 (6 - 26)
**Duration of residence in Germany**	
Less than 5 years	103 (2.7%)
5 to 10 years	115 (3.0%)
More than 10 years	124 (3.2%)
Born in Germany	3532 (91.2%)
**Household net income**^d^	
< 1.250	93 (2.4%)
1.250 - 2.249	397 (10.3%)
2.250 - 2.999	493 (12.8%)
3.000 - 3.999	1001 (26.0%)
4.000 - 4.999	960 (24.9%)
>5.000	914 (23.7%)
**Partnership status**	
Partner	3785 (97.7%)
No partner	89 (2.3%)
**Education**	
≤ 10 years	1008 (26.0%)
>10 years	2864 (73.9%)
**Current mental health problem**^e^	
Yes	274 (7.1%)
No	3460 (89.3%)
Don't know	140 (3.6%)
**Previous mental health problem**^e^	
Yes	977 (25.2%)
No	2793 (72.1%)
Don't know	104 (2.7%)
**Postpartum PTSD**	
CB-PTSD	143 (3.7%)
gPTSD	98 (2.5%)
comorbid CB-PTSD & gPTSD	15 (0.4%)
non-affected	3618 (93.4%)
**Barriers to help-seeking**	
Total barriers sum score^f^	31.5 ± 6.98 (15 - 60)
Fear regarding treatment and stigmatization^g^	10.16 ± 3.33 (5 - 25)
Health beliefs^g^	11.15 ± 2.8 (5 - 25)
Instrumental barriers^g^	10.2 ± 2.82 (5 - 23)

*n* (%) or *M* ± *SD* (Range). ^a^ total *N* varies slightly due to missing data, ^b^ in years, ^c^ in weeks, ^d^ in Euros per month and household, ^e^ based on self-report, ^f^ sum score of barriers, ^g^ subcategories of barriers

Among the total sample, 3.7% of women *(n* = 143) met diagnostic criteria for CB-PTSD, while 2.5% (*n* = 98) met diagnostic criteria for gPTSD. Due to the small sample size of women with comorbid CB-PTSD and gPTSD (*n* = 15), we excluded them from further data analyses. The majority of women (93.4%, *n* = 3,618) was not affected by postpartum PTSD. Table 2 compares predictors and outcomes across the three different groups revealing that only 1.4% of women with CB-PTSD self-reported their condition as PTSD. Self-reporting was slightly higher among women with gPTSD (5.1%), whereas 16 women reported PTSD despite not meeting diagnostic criteria. Half of the women with gPTSD had sought help for a mental health problem before, while only 35% of those with CB-PTSD had. Interestingly, women with CB-PTSD reported the highest total amount of barriers (M = 34.29, SD = 7.07), followed by women with gPTSD (M = 33.67, SD = 7.59), and non-affected women (M = 31.32, SD = 6.92). Likewise, women with CB-PTSD reported the highest barriers in all subcategories. Among all groups, barriers related to health beliefs were consistently higher than those related to fear regarding treatment and stigmatization as well as instrumental barriers. The three highest rated barriers among all women were: ‘I would rather talk to friends or family about my personal problems’ (*M* = 3.06, *SD* = 1.03), ‘I would not have childcare’ (*M* = 2.66, *SD* = 1.12), and ‘I would not have time for counseling or treatment’ (*M* = 2.51, *SD* = 1.03).

**Table 2:** Descriptive statistics of the predictors and dependent variables, subdivided into symptom groups

	CB-PTSD (*n* = 143)	gPTSD (*n* = 98)	non-affected (*n* = 3618)
**Self-report of PTSD**			
Yes	2 (1.4%)	5 (5.1%)	16 (0.4%)
No	141 (98.6%)	93 (94.9%)	3602 (99.6%)
**Knowledge of healthcare services**	8.58 ± 2.66 (1 - 13)	9.34 ± 2.56 (2 - 13)	8.97 ± 2.8 (0 - 13)
**Previous help-seeking**			
Yes	50 (35%)	49 (50%)	768 (21.2%)
No	93 (65%)	49 (50%)	2850 (78.8%)
**Previous experiences with help-seeking**			
Average - very good	44 (30.8%)	44 (44.9%)	715 (19.8%)
Negative	6 (4.2%)	4 (4.1%)	50 (1.4%)
No experiences	93 (65%)	50 (51%)	2853 (78.9%)
**Social support**	4.16 ± 0.54 (2.64 - 5)	4.09 ± 0.65 (1.86 - 4.93)	4.45 ± 0.49 (1.07 - 5)
**Severity of symptoms**			
City BiTS	19.92 ± 7.33 (7 - 41)	10.68 ± 8.96 (0 - 42)	5.05 ± 5.03 (0 - 40)
PC-PTSD-5	3.19 ± 2.63 (0 - 12)	10.41 ± 3.97 (4 - 20)	2.93 ± 2.31 (0 - 12)
**Household net income** ^a^	4.08 ± 1.41 (1 - 6)	3.75 ± 1.43 (1 - 6)	4.34 ± 1.35 (1 - 6)
**Perception of birth as traumatic** ^b^	6.92 ± 2.16 (0 - 10)	3.29 ± 3.02 (0 - 9)	2.92 ± 2.79 (0 - 10)
**Barriers to help-seeking**			
Total amount of barriers^c^	34.29 ± 7.07 (17 - 53)	33.67 ± 7.59 (17 - 51)	31.32 ± 6.92 (15 - 60)
Fear regarding treatment and stigmatization^d^	11.37 ± 3.57 (5 - 22)	11.01 ± 3.38 (5 - 21)	10.08 ± 3.3 (5 - 25)
Health beliefs^d^	11.58 ± 2.96 (5 - 20)	11.34 ± 3.13 (5 - 22)	11.12 ± 2.78 (5 - 25)
Instrumental barriers^d^	11.34 ± 2.9 (5 - 21)	11.33 ± 3.21 (5 - 19)	10.12 ± 2.78 (5 - 23)

*n* (%) or *M* ± *SD* (Range). ^a^ divided into six categories, ^b^ self-rated on a scale range from 0 (‘not at all traumatic’) to 10 (‘very traumatic’), ^c^ sum score of barriers, ^d^ subcategories of barriers

Moreover, women with CB-PTSD rated their birth experience as most traumatic (*M* = 6.92, *SD* = 2.16). Minor differences were observed in predictors such as knowledge of healthcare services, social support, and household net income.

### Associations between predictors and the total amount of barriers to help-seeking

**Fig. 4:**
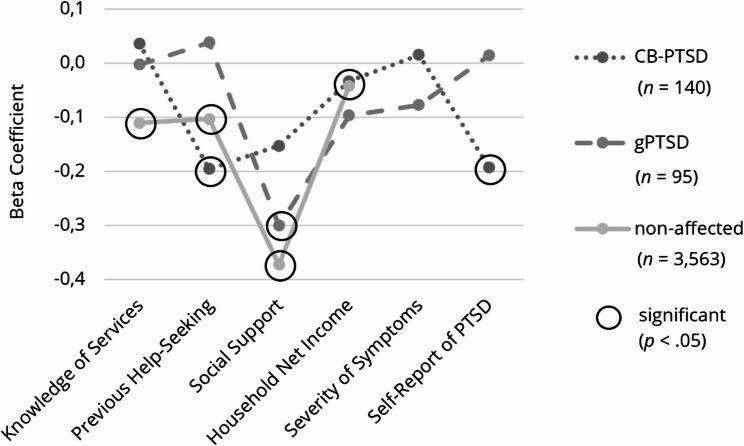
Effect Sizes of the Predictors on the Total Amount of Barriers to Help-Seeking, Subdivided into Symptom Groups**. **Illustration of the results, subdivided into symptom groups: childbirth-related PTSD (CB-PTSD) and general PTSD (gPTSD)

Three multiple linear regression analyses were conducted to examine the associations between predictors (knowledge of healthcare services, previous help-seeking, social support, household net income, severity of symptoms, self-report of PTSD) and the total amount of barriers to help-seeking in each group. We excluded 20 outliers from subsequent analyses, along with 61 women with missing values for the barrier sum score. The regression model significantly predicted barriers among women with CB-PTSD, (*R*^*2*^ = 0.096, adjusted *R*^*2*^ = 0.056, *F* (6,134) = 2.382, *p* <.05) and non-affected women (*R*^*2*^ = 0.171, adjusted *R*^*2*^ = 0.170, *F* (4, 3559) = 183.487, *p* <.001), but not among women with gPTSD (*R*^*2*^ = 0.111, adjusted *R*^*2*^ = 0.051, *F* (6, 89) = 1.856, *p* =.097). To compare effect sizes among groups, we conducted a visual analysis (see Fig. 4).

Among women with CB-PTSD, previous help-seeking (*ß* = − 0.196, *p* <.05) and self-reported PTSD (*ß* = − 0.193, *p* <.05) predicted lower barriers to help-seeking. In women with gPTSD, higher social support predicted lower barriers (*ß* = − 0.301, *p* <.05), whereas among non-affected women, greater knowledge of healthcare services (*ß* = − 0.111, *p* <.001), previous help-seeking (*ß* = − 0.104, *p* <.001), higher social support (*ß* = − 0.373, *p* <.001), and higher household net income (*ß* = − 0.042, *p* <.05) predicted lower barriers to help-seeking. However, negative experiences in previous help-seeking predicted higher barriers (*ß* = 0.167, *p* <.001), as shown in Table 7. All effect sizes, except for social support, were small according to Cohen’s classification [56]. For further information, please refer to Table 3.

**Table 3:** Multiple linear regression analyses of the total amount of barriers to help-seeking, subdivided into symptom groups

	*Women with CB-PTSD*					*CI*		*Women with gPTSD*					CI	
Predictors	B	SE	β	*t*	*p*	LL	UL	B	SE	β	*t*	*p*	LL	UL
(Constant)	43.108	5.393		7.993	<.001	32.441	53.775	51.303	6.458		7.944	<.001	38.472	64.134
Self-Report of PTSD	−11.315	4.884	**-.193**	−2.317	.022	−20.974	−1.656	0.493	3.580	.014	0.138	.891	−6.621	7.607
Knowledge of Services	0.095	0.224	.036	0.425	.671	-0.348	0.539	-0.010	0.316	-.003	-0.030	.976	-0.637	0.618
Previous Help-Seeking	−2.853	1.243	**-.196**	−2.295	.023	−5.311	-0.395	0.579	1.619	.038	0.358	.721	−2.638	3.796
Social Support	−1.969	1.103	-.153	−1.785	.077	−4.151	0.213	−3.516	1.175	**-.301**	−2.994	.004	−5.850	−1.182
Severity of Symptoms	0.015	0.084	.015	0.173	.863	-0.152	0.181	-0.151	0.193	-.078	-0.780	.437	-0.534	0.233
Household Net Income	-0.168	0.416	-.034	-0.403	.687	-0.991	0.656	-0.515	0.539	-.097	-0.955	.342	−1.585	0.556
	Non-affected women					CI								
Predictors	B	SE	β	*t*	*p*	LL	UL							
(Constant)	57.917	1.001		57.886	<.001	55.955	59.878							
Knowledge of Services	-0.269	0.037	**-.111**	−7.190	<.001	-0.343	-0.196							
Previous Help-Seeking	−1.724	0.257	**-.104**	−6.722	<.001	−2.227	−1.221							
Social Support	−5.163	0.219	**-.373**	−23.610	<.001	−5.592	−4.734							
Household Net Income	-0.211	0.078	**-.042**	−2.721	.007	-0.364	-0.059							

*Dependent variable *Total amount of barriers to help-seeking, *B *Unstandardized coefficient, *SE *Standard error, *ß *Standardized coefficient, *CI *Confidence interval, *LL *Lower level, *UL *Upper level

### Associations between predictors and subcategories of barriers to help-seeking

#### Fear regarding treatment and stigmatization

**Fig. 5:**
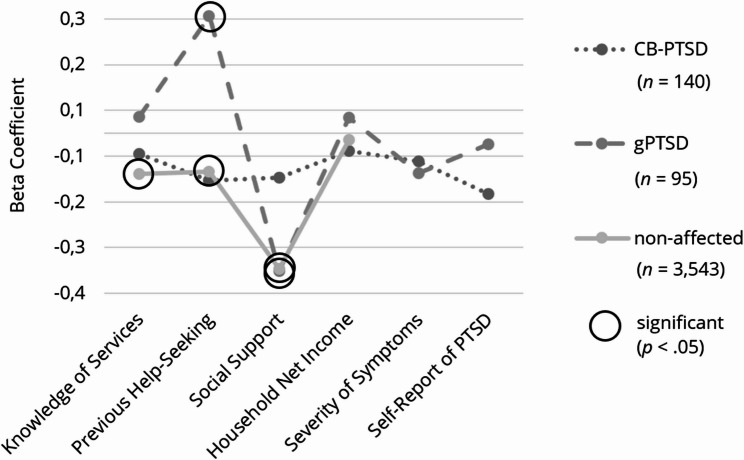
Effect Sizes of the Predictors on Fear Regarding Treatment and Stigmatization, Subdivided into Symptom Groups. Note. Illustration of the results, subdivided into symptom groups: childbirth-related PTSD (CB-PTSD) and general PTSD (gPTSD)

To analyze the associations between the predictors and fear regarding treatment and stigmatization, we excluded 44 outliers and 81 women due to missing values on this subscale. In the CB-PTSD group, the overall regression model did not reach significance (*R*^*2*^ = 0.049, adjusted *R*^*2*^ = 0.006, *F* (6, 134) = 1.147, *p* =.339), with no factor predicting fear regarding treatment and stigmatization. However, the overall models were significant for the gPTSD group (*R*^*2*^ = 0.167, adjusted *R*^*2*^ = 0.111, *F* (6, 89) = 2.971, *p* <.05) and non-affected women (*R*^*2*^ = 0.105, adjusted *R*^*2*^ = 0.104, *F* (4, 3539) = 104.018, *p* <.001). In women with gPTSD, higher social support predicted lower fear (*ß* = − 0.302, *p* <.05), while previous help-seeking predicted higher fear (*ß* = 0.256, *p* <.05). We conducted a sub-analysis to examine if negative experiences in previous help-seeking contributed to this association, but results did not support this assumption. Among non-affected women, greater knowledge of healthcare services (*ß* = − 0.089, *p* <.001), higher social support (*ß* = − 0.296, *p* <.001), and previous help-seeking (*ß* = − 0.084, *p* <.001) significantly predicted lower fear. In addition, negative experiences in previous help-seeking predicted higher fear regarding treatment and stigmatization (*ß* = 0.110, *p* <.05), as detailed in Table 7. Apart from the moderate effect of social support in women with gPTSD, all significant effects were small in size (see Fig. 5). For further details, please refer to Table 4.

**Table 4:** Multiple linear regression analyses of the barrier subcategory fear regarding treatment and stigmatization, subdivided into symptom groups

	Women with CB-PTSD					CI		Women with gPTSD					CI	
Predictors	B	SE	β	*t*	*p*	LL	UL	B	SE	β	*t*	*p*	LL	UL
(Constant)	15.611	2.745		5.688	<.001	10.183	21.039	16.626	2.777		5.986	<.001	11.107	22.144
Self-Report of PTSD	−3.842	2.484	-.132	−1.547	.124	−8.756	1.071	-0.366	1.540	-.024	-0.238	.813	−3.425	2.694
Knowledge of Services	-0.058	0.114	-.045	-0.514	.608	-0.283	0.166	0.048	0.136	.036	0.352	.726	-0.222	0.318
Previous Help-Seeking	-0.748	0.632	-.104	−1.184	.239	−1.997	0.502	1.725	0.696	**.256**	2.477	.015	0.341	3.108
Social Support	-0.618	0.562	-.097	−1.100	.273	−1.730	0.493	−1.564	0.505	**-.302**	−3.095	.003	−2.567	-0.560
Severity of Symptoms	-0.029	0.042	-0.061	-.682	.496	-0.111	0.054	-0.074	0.083	-.087	-0.895	.373	-0.239	0.091
Household Net Income	-0.094	0.210	-.038	-0.445	.657	-0.510	0.323	0.081	0.232	.034	0.350	.727	-0.379	0.542
	Non-affected women					CI								
Predictors	B	SE	β	*t*	*p*	LL	UL							
(Constant)	19.538	0.481		40.661	<.001	18.596	20.480							
Knowledge of Services	-0.099	0.018	**-.089**	−5.540	<.001	-0.135	-0.064							
Previous Help-Seeking	-0.638	0.123	**-.084**	−5.188	<.001	-0.880	-0.397							
Social Support	−1.885	0.105	**-.296**	−17.981	<.001	−2.091	−1.679							
Household Net Income	-0.035	0.037	-.015	-0.953	.341	-0.108	0.038							

*Dependent variable *Subcategory fear regarding treatment and stigmatization, *B *unstandardized coefficient, *SE *standard error, *ß *standardized coefficient, *CI *confidence interval, *LL *lower level, *UL *Upper level

#### Health beliefs

**Fig. 6:**
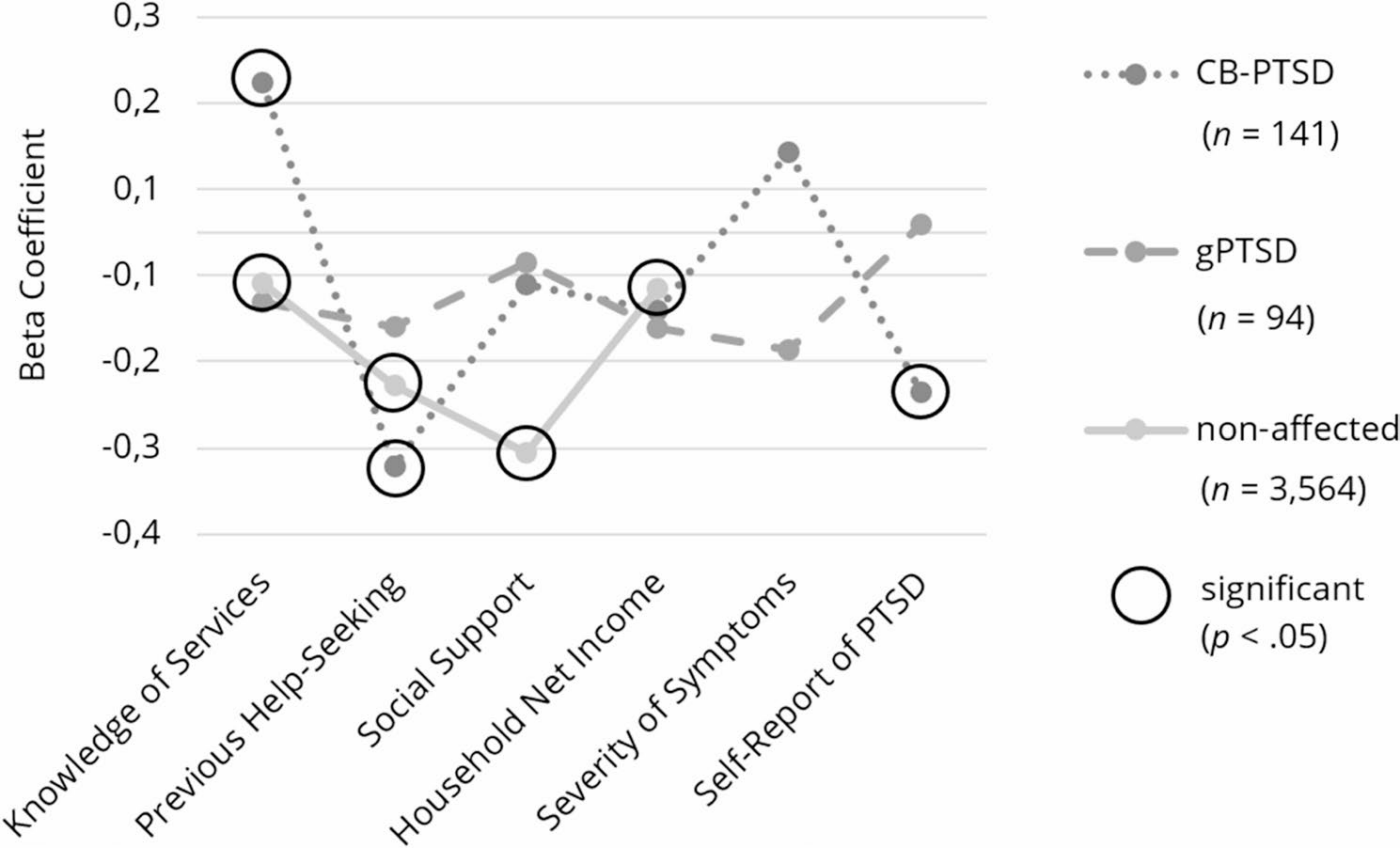
Effect Sizes of the Predictors on Health Beliefs, Subdivided into Symptom Groups. Illustration of the results, subdivided into symptom groups: childbirth-related PTSD (CB-PTSD) and general PTSD (gPTSD)

To analyze the associations between the predictors and barriers related to health beliefs, we excluded 18 outliers and 60 women due to missing values on this subscale. The regression models were significant in the CB-PTSD group (*R*^*2*^ = 0.135, adjusted *R*^*2*^ = 0.096, *F* (6, 135) = 3.502, *p* <.05) and among non-affected women (*R*^*2*^ = 0.102, adjusted *R*^*2*^ = 0.101, *F* (4, 3560) = 100.584, *p* <.001). However, they were not significant among women with gPTSD (*R*^*2*^ = 0.052, adjusted *R*^*2*^ = − 0.013, *F* (6, 88) = 0.803, *p* =.571), with no factor predicting health beliefs. Among women with CB-PTSD, self-report of PTSD (*ß* = − 0.186, *p* <.05) and previous help-seeking (*ß* = − 0.271, *p* <.05) predicted lower barriers, whereas greater knowledge of healthcare services predicted higher barriers (*ß* = 0.173, *p* <.05). Among non-affected women, greater knowledge of healthcare services (*ß* = − 0.059, *p* <.001), previous help-seeking (*ß* = − 0.178, *p* <.001), higher social support (*ß* = − 0.256, *p* <.001), and higher household net income (*ß* = − 0.065, *p* <.001) predicted lower barriers. In contrast, a sub-analysis revealed that negative experiences in previous help-seeking predicted higher barriers in non-affected women (*ß* = 0.166, *p* <.001). All predictors had small effect sizes in the health beliefs subcategory (see Fig. 6). For more detailed information, please refer to Tables 5 and 7.

**Table 5:** Multiple Linear Regression Analyses of the Barrier Subcategory Health Beliefs, Subdivided into Symptom Groups

	Women with CB-PTSD					CI		Women with gPTSD					CI	
Predictors	B	SE	β	*t*	*p*	LL	UL	B	SE	β	*t*	*p*	LL	UL
(Constant)	11.978	2.244		5.337	<.001	7.539	16.416	14.971	2.587		5.788	<.001	9.831	20.112
Self-Report of PTSD	−4.664	2.032	**-.186**	−2.296	.023	−8.682	-0.646	0.115	1.435	.009	0.080	.936	−2.736	2.966
Knowledge of Services	0.193	0.093	**.173**	2.082	.039	0.010	0.377	-0.095	0.129	-.081	-0.735	.464	-0.350	0.161
Previous Help-Seeking	−1.675	0.515	**-.271**	−3.255	.001	−2.693	-0.657	-0.645	0.657	-.110	-0.982	.329	−1.950	0.660
Social Support	-0.326	0.459	-.060	-0.710	.479	−1.234	0.582	-0.157	0.470	-.035	-0.335	.739	−1.092	0.777
Severity of Symptoms	0.038	0.034	.093	1.107	.270	-0.030	0.105	-0.102	0.077	-.137	−1.321	.190	-0.256	0.052
Household Net Income	-0.191	0.172	-.091	−1.109	.269	-0.531	0.149	-0.231	0.218	-.111	−1.060	.292	-0.664	0.202
	Non-affected women					CI								
Predictors	B	SE	β	*t*	*p*	LL	UL							
(Constant)	18.771	0.420		44.682	<.001	17.947	19.594							
Knowledge of Services	-0.057	0.016	**-.059**	−3.629	<.001	-0.088	-0.026							
Previous Help-Seeking	−1.189	0.108	**-.178**	−11.021	<.001	−1.400	-0.977							
Social Support	−1.427	0.092	**-.256**	−15.525	<.001	−1.608	−1.247							
Household Net Income	-0.130	0.033	**-.065**	−3.993	<.001	-0.194	-0.066							

*Dependent variable *Subcategory health beliefs, *B *Unstandardized coefficient, *SE *Standard error, *ß *Standardized coefficient, *CI *Confidence interval, *LL *Lower level, *UL *Upper level

#### Instrumental barriers

**Fig. 7:**
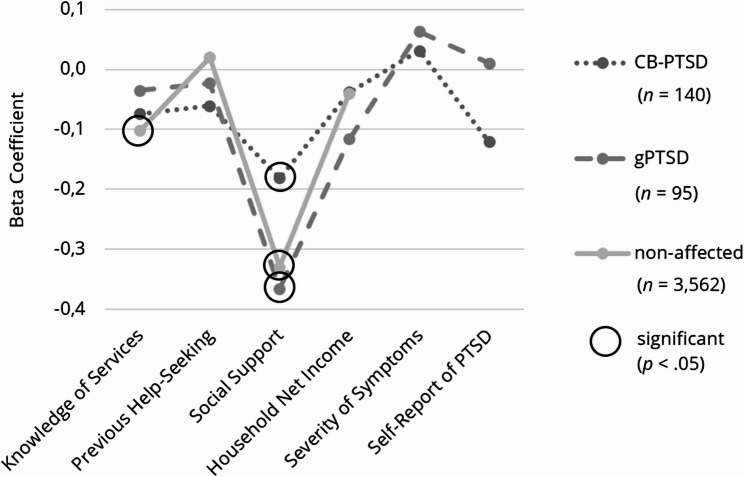
Effect Sizes of the Predictors on Instrumental Barriers, Subdivided into Symptom Groups. Illustration of the results, subdivided into symptom groups: childbirth-related PTSD (CB-PTSD) and general PTSD (gPTSD)

To analyze the associations of the predictors and instrumental barriers, we excluded 22 outliers and 62 women due to missing values on this subscale. The regression model did not reach significance in the CB-PTSD group (*R*^*2*^ = 0.064, adjusted *R*^*2*^ = 0.022, *F* (6, 134) = 1.519, *p* =.176), unlike the models of women with gPTSD (*R*^*2*^ = 0.152, adjusted *R*^*2*^ = 0.095, *F* (6, 89) = 2.667, *p* <.05) and non-affected women (*R*^*2*^ = 0.136, adjusted *R*^*2*^ = 0.135, *F* (4, 3558) = 139.957, *p* <.001). Among women with CB-PTSD, higher social support significantly predicted lower instrumental barriers (*ß* = − 0.181, *p* <.05), whereas previous negative experiences with help-seeking predicted higher instrumental barriers (*ß* = 0.289, *p* <.05). Likewise, higher social support significantly predicted lower instrumental barriers among women with gPTSD (*ß* = − 0.366, *p* <.001). Among non-affected women, higher social support (*ß* = − 0.330, *p* <.001) and greater knowledge of healthcare services (*ß* = − 0.102, *p* <.001) significantly predicted lower instrumental barriers. In contrast, previous negative experiences with help-seeking predicted higher instrumental barriers for non-affected women (*ß* = 0.119, *p* <.001). The effect sizes of social support among women with gPTSD and non-affected women were moderate, while the other effect sizes were small (see Fig. 7). For further information, please refer to Tables 6 and 7.

**Table 6:** Multiple linear regression analyses of the barrier subcategory instrumental barriers, subdivided into symptom groups

	Women with CB-PTSD					CI		Women with gPTSD					CI	
Predictors	B	SE	β	*t*	*p*	LL	UL	B	SE	β	*t*	*p*	LL	UL
(Constant)	15.466	2.210		6.997	<.001	11.094	19.838	19.970	2.654		7.525	<.001	14.697	25.243
Self-Report of PTSD	−2.863	2.001	-.121	−1.430	.155	−6.821	1.096	0.900	1.471	.063	0.612	.542	−2.023	3.824
Knowledge of Services	-0.078	0.092	-.074	-0.847	.398	-0.260	0.104	-0.044	0.130	-.035	-0.340	.735	-0.302	0.214
Previous Help-Seeking	-0.358	0.509	-.061	-0.703	.483	−1.366	0.649	-0.149	0.665	-.023	-0.225	.823	−1.471	1.173
Social Support	-0.938	0.452	**-.181**	−2.074	.040	−1.832	-0.044	−1.796	0.483	**-.366**	−3.721	<.001	−2.755	-0.837
Severity of Symptoms	0.012	0.035	.031	0.349	.728	-0.056	0.080	0.008	0.079	.010	0.107	.915	-0.149	0.166
Household Net Income	0.075	0.171	.038	0.441	.660	-0.262	0.413	-0.260	0.221	-.116	−1.175	.243	-0.700	0.180
	Non-affected women					CI								
Predictors	B	SE	β	*t*	*p*	LL	UL							
(Constant)	19.350	0.407		47.534	<.001	18.552	20.148							
Knowledge of Services	-0.099	0.015	**-.102**	−6.458	<.001	-0.129	-0.069							
Previous Help-Seeking	0.128	0.104	.020	1.231	.218	-0.076	0.333							
Social Support	−1.816	0.089	**-.330**	−20.429	<.001	−1.990	−1.641							
Household Net Income	-0.079	0.032	-.040	−2.497	.013	-0.141	-0.017							

Dependent variable Subcategory instrumental barriers, *B *Unstandardized coefficient, *SE *Standard error, *ß *Standardized coefficient, *CI *Confidence interval, *LL *Lower level, *UL *Upper level

**Table 7:** Multiple linear regression sub-analysis of the predictor negative experiences with previous help-seeking, subdivided into symptom groups

	Women with CB-PTSD					Women with gPTSD					Non-affected women				
Predictor	B	SE	β	*t*	*p*	B	SE	β	*t*	*p*	B	SE	β	*t*	*p*
(Constant)	32.159	1.053		30.531	0.000	33.767	1.143		29.545	0.000	30.366	0.268		113.315	0.000
Neg. experiences	3.508	3.041	0.164	1.154	0.254	5.233	3.918	0.195	1.336	0.188	4.891	1.048	**0.167**	4.669	0.000
*Note. *Dependent variable = total amount of barriers to help-seeking															
(Constant)	10.705	0.531		20.151	0.000	11.698	0.546		21.444	0.000	9.773	0.129		75.787	0.000
Neg. experiences	0.462	1.533	0.043	0.301	0.764	2.052	1.870	0.161	1.098	0.278	1.547	0.504	**0.110**	3.069	0.002
*Note. *Dependent variable = subcategory fear regarding treatment and stigmatization															
(Constant)	10.659	0.437		24.395	0.000	10.698	0.447		23.950	0.000	10.285	0.107		95.686	0.000
Neg. experiences	0.674	1.261	0.077	0.535	0.595	2.802	1.531	0.263	1.830	0.074	1.950	0.420	**0.166**	4.645	0.000
*Note. *Dependent variable = subcategory health beliefs															
(Constant)	10.795	0.393		27.478	0.000	11.372	0.486		23.406	0.000	10.311	0.107		96.097	0.000
Neg. experiences	2.371	1.134	**0.289**	2.091	0.042	0.378	1.665	0.034	0.227	0.822	1.389	0.419	**0.119**	3.313	0.001

*Dependent variable *Subcategory instrumental barriers, *neg. experiences *Negative experiences with previous help-seeking, *B *Unstandardized coefficient, *SE *Standard error, *ß *Standardized coefficient

#### Power analysis

To determine the statistical power of the regression analyses, we computed post-hoc power analyses using G*Power 3.1.9.7 [57] for F-tests (linear multiple regression). In the group of women with CB-PTSD, the sample achieved 94.59% power (1-beta), based on an effect size of *f*^*2*^ = 0.02, a group size of *n* = 143, and a significance level of α = 0.05. Lower power was observed in the group of women with gPTSD, with a sample size of *n* = 98 and an achieved power of 80.35%. In the group of non-affected women, we attained 100% power with a sample size of *n* = 3,618. These findings suggest that our sample size was sufficient to reliably detect small effects in all groups.

## Discussion

This study aimed to investigate strategies to reduce barriers to help-seeking among postpartum women affected by CB-PTSD, gPTSD, and non-affected women. In our sample, 3.7% of women met criteria for CB-PTSD, 2.5% for gPTSD, and 0.4% for comorbid CB-PTSD and gPTSD, while 92.6% did not experience clinically significant symptoms of postpartum PTSD. These prevalence rates are higher than previously assessed rates of 5.44% for women with postpartum PTSD [5] and lower than those of 4.7% for women with CB-PTSD [6]. Our findings highlight the critical role of social support in reducing barriers, particularly among women with gPTSD. Recognizing one’s PTSD, on the other hand, emerges as crucial for overcoming barriers among women with CB-PTSD. While previous help-seeking behavior may reduce barriers related to health beliefs among women with CB-PTSD, it may increase fear regarding treatment and stigmatization among those with gPTSD. Greater knowledge of healthcare services may decrease barriers for non-affected women, but it may increase barriers related to health beliefs for women with CB-PTSD. Household net income predicted lower barriers only among non-affected women, while severity of symptoms showed no significant association with barriers. In sum, our findings underscore the complex nature of help-seeking behaviors among postpartum women.

### Barriers across the symptom groups

In our descriptive statistics analysis, we observed higher barriers to help-seeking among women with postpartum PTSD compared to non-affected women, encompassing both the total amount and all subcategories of barriers. This is in line with statistical analyses on group differences within our sample, conducted by Jehn et al. [48], revealing that women with CB-PTSD are particularly vulnerable to encountering higher barriers to help-seeking. This difference may be attributed to the fact that while non-affected women reported their barriers to help-seeking, they were asked to imagine being affected by a mental health disorder. Consequently, their responses may not accurately reflect but rather underestimate the barriers faced by affected women. This aligns with unpublished observations from Seefeld et al. [47] on women with PPD and PAD highlighting the challenge non-affected women encounter in empathizing with affected women. Therefore, our primary focus will be on barriers affected women face. In the following section, we outline and discuss our findings on the associations between predictors and barriers to help-seeking.

### Associations between predictors and barriers to help-seeking

#### Social support

Among all women, higher levels of social support were significantly associated with lower instrumental barriers, which could be attributed to items such as ‘I would not have childcare’ in this subscale. The lack of childcare as a barrier has been previously assessed by Boyd et al. [34], emphasizing the crucial role of supportive individuals in aiding postpartum women. These individuals can provide practical assistance by offering childcare during treatment sessions, thereby reducing barriers to help-seeking. Among women with gPTSD and non-affected women, having higher social support was associated with a lower total amount of barriers to help-seeking, as well as lower fear regarding treatment and stigmatization. This aligns with previous studies on PPD, where women reported that support and encouragement from friends and family increased their trust in healthcare providers and treatment and thereby reduced barriers [30, 32, 33, 58, 59]. However, among women affected by CB-PTSD, this association was not found. This discrepancy may be attributed to the relatively recent onset of symptoms, leading to their misinterpretation as normal aspects of motherhood [36, 60]. Consequently, friends and family may offer emotional and practical support rather than actively promoting professional help-seeking [29, 61, 62].

#### Self-report of PTSD

We found that self-reporting one’s PTSD predicted a lower total amount of barriers as well as lower barriers related to health beliefs among women with CB-PTSD. This vital role of recognizing and identifying one’s symptoms in order to seek help aligns with prior research [35, 63]. In our sample, only 1.4% of women with CB-PTSD were able to identify their PTSD. The remaining women may struggle to distinguish between normal transitions to motherhood and symptoms of PTSD, a phenomenon also observed in women with PPD [26, 36]. Conversely, this effect was not found among women with gPTSD, possibly due to the different onset of symptoms. We assume that women with gPTSD have often encountered symptoms before and unrelated to childbirth, potentially reducing their likelihood of misinterpreting them as typical postpartum experiences. Nevertheless, even among women with gPTSD, self-reporting PTSD remained low (5.1%). This aligns with previous findings [63, 64], indicating that PTSD often goes unrecognized by the affected individuals, regardless of the underlying traumatic event.

Furthermore, 0.4% of women in our sample reported to suffer from PTSD without meeting diagnostic criteria. This discrepancy may reflect diagnostic thresholds excluding subclinical postpartum stressors. As diagnostic systems rely on categorical classifications, they do not adequately reflect the dimensional nature of psychological distress. However, such posttraumatic stress (PTSS) has been observed in up to 12.3% of postpartum women [6]. Therefore, a context-sensitive diagnostic approach is essential to ensure care for women with trauma-related symptoms. Failure to acknowledge subthreshold cases may result in feelings of invalidation and reduced help-seeking.

#### Previous help-seeking

Another noteworthy finding was that among women with CB-PTSD and non-affected women, those who had sought help for mental health problems before reported lower barriers to seeking help again. This was found for both the total amount of barriers and those related to health beliefs, aligning with prior research on postpartum women [38, 65–67]. Conversely, among women with gPTSD, previous help-seeking was identified as a risk factor, with increased fear regarding treatment and stigmatization observed in those who had sought help before. The assumption that this discrepancy may be attributed to negative experiences in previous help-seeking was not confirmed in a sub-analysis. However, it is important to highlight that only a small number of women with gPTSD (4 out of 49) reported negative experiences, which may limit the statistical power of the sub-analysis.

#### Knowledge of healthcare services

Among non-affected women, having greater knowledge of healthcare services was associated with reduced barriers across all subcategories. However, this was contrary among women with CB-PTSD, where greater knowledge was linked to increased barriers concerning health beliefs. This finding contradicts numerous studies focusing on postpartum women with mental health issues [26, 33, 37, 61]. Nevertheless, it aligns with a study by Silva et al. [59] on women with PPD suggesting that knowledge limitations regarding treatment options do not significantly impact women’s help-seeking behavior. Likewise, it implies that while non-affected women may perceive a lack of knowledge as a significant barrier for affected women, the reality for women with CB-PTSD differs. However, psychoeducation is not considered harmful; on the contrary, it is likely to support affected women in gaining a clearer understanding of when their symptoms reflect a PTSD and when they do not [12]. Nonetheless, it is possible that women who are well-informed about available services may be particularly aware that the current healthcare system in Germany is not yet adequately equipped to address the specific needs of women with CB-PTSD. Many services remain primarily focused on PPD. As a result, women with CB-PTSD may report high barriers related to health beliefs, as they may assume that the services available will not adequately address their specific concerns and symptoms.

#### Household net income

Among non-affected women, a higher household net income was found to predict lower barriers, particularly those related to health beliefs. This association was not found among women with postpartum PTSD, which contrasts with prior international studies [38, 68]. Such discrepancy may be attributed to the characteristics of our sample. The majority of women reported a household net income exceeding 3,000 Euro per month and lived in Dresden, Germany. As a result, they benefited from free healthcare provided by public health insurance as well as accessible healthcare services, effectively reducing travel expenses. An explanation for the presence of significant results among non-affected women may be the large sample size within this group (*n* = 3,618), resulting in greater statistical power. Consequently, even small effect sizes may become statistically significant (Lantz, 2013).

#### Severity of symptoms

The severity of symptoms did not predict barriers to help-seeking among affected women. This aligns with a finding from Jehn et al. [48], indicating that symptom severity is not associated with women’s likelihood to seek help. However, prior research on the influence of symptom severity on help-seeking behavior is ambiguous. Studies on PPD suggest that higher symptom severity may lower barriers by increasing the urgency to seek help [65, 69–71]. Conversely, research on veterans with PTSD suggests the opposite effect, with higher severity decreasing energy, thereby increasing barriers [40]. Adding to the complexity, unpublished research from Seefeld et al. [47] found that among women with PPD, greater symptom severity predicted a reduced likelihood of seeking help, whereas among women with PAD, it predicted a higher likelihood of help-seeking. Our findings do not support either direction. This may be due to our criteria for group allocation in using a cutoff of at least 4 existing symptoms of PTSD. Consequently, all women who met criteria automatically had a relatively high severity of symptoms; otherwise, they were classified as non-affected. Furthermore, it is worth noting that we conducted interviews 3–4 months after obtaining women’s consent to participate, and some women may have dropped out due to highly severe symptoms, impacting our findings. These reasons may explain the absence of observed effects in our results, as well as in those of Jehn et al. [48], who used the same dataset.

### Strengths and limitations

While previous studies have primarily focused on women affected by PPD, our study used a large sample of women to examine PTSD in the postpartum period. Moreover, we made a clear distinction between cases of postpartum PTSD related to childbirth and those related to other traumatic events. To ensure the robustness of our findings and to assess symptoms accurately, we used validated instruments. Additionally, we benefited from a high response rate in our study, which allowed drawing valid conclusions, and we introduced a new approach to investigating barriers to help-seeking by identifying factors that may increase or decrease such barriers. As a result, our research has the potential to guide policy decisions and inform healthcare services about the steps that need to be taken to reduce barriers to help-seeking among postpartum women.

In addition to these strengths, it is important to acknowledge the limitations of our study, which may affect the generalizability of the results. First, our data collection method via telephone interviews may have been influenced by social desirability [72], potentially compromising the accuracy of responses. Moreover, this method may have contributed to a selection bias, as severely affected women may not have had the capacity to participate in a long telephone interview. Additionally, the majority of women in our sample had a household net income exceeding the average in Dresden [73] and resided in Germany, thereby limiting the broader applicability of our results to all postpartum women. Furthermore, the relatively small number of women in our sample who reported negative experiences in previous help-seeking may constrain the validity of our sub-analysis. Another constraint is the absence of statistical tests to assess significant group differences in demographic data. The descriptive analyses limit the ability to draw definitive conclusions about the role of demographic variables in the observed outcomes. Lastly, the reliability analyses revealed low internal consistency for certain barrier subscales, particularly health beliefs (α = 0.64) and instrumental barriers (α = 0.58). Therefore, it is important to interpret the corresponding results with caution.

### Research implications

In this work, we have identified significant factors that may reduce barriers to help-seeking among women with postpartum PTSD. However, we assume that there are additional factors predicting barriers, which we were unable to analyze with the available data. Consequently, further investigation into additional predictors is necessary. Potential factors to examine include self-efficacy, attitudes toward help-seeking, and perceived need for treatment, all of which have been identified in previous research as potential barriers to help-seeking among women experiencing PPD [65, 69, 71, 74–77]. Moreover, prior studies have shown that women from ethnic minority backgrounds and migrant populations are at increased risk of developing mental health problems. Despite this elevated risk, many women do not seek help for their symptoms, as healthcare services may not adequately address the specific needs of migrant women [38, 60, 76]. Therefore, ethnic identity and country of origin represent additional predictors requiring further exploration. Additionally, conducting the same analyses with a larger sample of women affected by comorbid CB-PTSD and gPTSD may provide a more comprehensive understanding of their specific barriers to help-seeking. Beyond, future research could investigate whether the prevalence of self-reported CB-PTSD is higher when assessments are conducted at a later stage of the postpartum period, as increased emotional distance may facilitate an accurate identification of PTSD symptoms. Finally, given the cross-sectional nature of the INVITE study, drawing causal conclusions is not possible. Conducting follow-up interviews extending beyond the 6-month postpartum period could offer a more comprehensive understanding of changes in help-seeking behaviors and factors influencing such changes.

### Practical implications

Given the significant adverse outcomes associated with postpartum PTSD, it is imperative to implement strategies to prevent it from occurring in the first place. Midwives and psychologists can play a key role in this by providing psychosocial support and implementing prevention and intervention strategies for women with traumatic birth experiences [6, 78]. Psychoeducation may be beneficial in helping women recognize emotional changes and symptoms within themselves, thus encouraging help-seeking behavior [79]. Additionally, it becomes imperative that women encounter minimal barriers to access mental healthcare. In our study, we found that only a minority of affected women were aware of their condition and therefore self-reported it. However, among those with CB-PTSD who did self-report it, we observed lower barriers to help-seeking. Especially when considering that women with CB-PTSD are more vulnerable to facing higher barriers to help-seeking [48], it becomes a crucial component to support these women in recognizing their symptoms. One potential approach to achieve this is to implement PTSD screenings for all postpartum women, similar to what has partially been implemented for PPD [80]. These screenings could easily be integrated, for instance, during routine preventive medical check-ups for children, using short instruments such as the PC-PTSD-5 [44] and the City BiTS [42, 43]. Furthermore, healthcare professionals could play a vital role in identifying symptoms of PTSD among women by asking about their childbirth experience [81]. Moreover, given that instrumental barriers are notably higher among women with postpartum PTSD compared to non-affected women [48], we found that such barriers can be overcome by providing sufficient social support for affected women. This can be achieved by equipping family and friends with comprehensive information about women’s needs, enabling them to offer practical support as well as to encourage professional help-seeking. For single mothers and women in vulnerable family situations, initiatives such as home-based midwifery care [82] could be expanded. Furthermore, our research revealed that previous help-seeking can lead to increased stigmatization and fear of treatment, which may prevent women from seeking help for future problems. It is therefore essential that women have positive experiences with healthcare services, that these are sensitive to women’s needs and adapted to their preferences [83]. While existing services predominantly focus on PPD, greater emphasis should be placed on the support of women affected by postpartum PTSD [12, 84]. Additionally, to reduce stigmatization, educational campaigns could be developed aimed at addressing knowledge gaps regarding postpartum mental health issues. With support and funding from governmental institutions for such campaigns, a greater awareness of postpartum PTSD within society could be achieved. Ultimately, this may increase understanding and empathy for affected women, thereby encouraging them to seek help.

## Conclusions

Postpartum PTSD poses a significant challenge with far-reaching consequences, not only for affected women but also their social surroundings. Although first effective treatment options have been identified, only a small proportion of affected women seek professional help for their symptoms. This study aimed to enhance the understanding of the reasons preventing women from seeking help. Through an examination of factors that may increase or decrease barriers to help-seeking, we found significant associations that could help to overcome such barriers. For instance, our findings highlight the critical role of social support in reducing barriers, particularly among women with gPTSD. Recognizing one’s symptoms of PTSD, on the other hand, emerges as crucial for reducing barriers among women with CB-PTSD. Further, while previous help-seeking behavior may reduce barriers among women with CB-PTSD, it may increase them among women with gPTSD. Moreover, although greater knowledge of healthcare services may potentially decrease barriers for non-affected women, it may increase barriers among women with CB-PTSD. Last, household net income may reduce barriers among non-affected women, while severity of symptoms does not seem to predict barriers. Implementing our findings into practice could involve screenings of women for mental health issues during the postpartum period, thereby assisting them to identify potential symptoms. Additionally, offering psychoeducation could equip affected women and their social surroundings with comprehensive information about women’s emotional changes within the postpartum period as well as their specific needs. Furthermore, it remains crucial to raise awareness within society about postpartum mental health issues, for instance, using educational campaigns. Such initiatives can contribute to reducing stigmatization while increasing empathy for affected women. It is our hope that these recommendations may be implemented, thereby assisting women in their return to healthy functioning and an improved quality of life following trauma.

## Data Availability

The dataset presented in this article is not available to the public due to legal and ethical restrictions. The release of participant data was not included in the informed consent for the study. Requests for access to the datasets should be addressed to the project managers Susan Garthus-Niegel and Julia Schellong.
